# Potassium management with finerenone: Practical aspects

**DOI:** 10.1002/edm2.360

**Published:** 2022-09-15

**Authors:** Christoph Wanner, Paola Fioretto, Csaba P. Kovesdy, Jolanta Malyszko, Roberto Pecoits‐Filho, Oliver Schnell, Patrick Rossignol

**Affiliations:** ^1^ Medical University of Würzburg Würzburg Germany; ^2^ Department of Medicine University of Padova Padova Italy; ^3^ Department of Medicine University of Tennessee Health Science Center Memphis Tennessee USA; ^4^ Department of Nephrology, Dialysis and Internal Medicine Medical University of Warsaw Warsaw Poland; ^5^ School of Medicine Pontifical Catholic University of Paraná Curitiba Brazil; ^6^ DOPPS Program Area, Arbor Research Collaborative for Health Ann Arbor Michigan USA; ^7^ Sciarc GmbH Baierbrunn Germany; ^8^ Forschergruppe Diabetes e. V. Neuherberg (Munich) Germany; ^9^ Université de Lorraine INSERM CIC‐P 1433, CHRU de Nancy, INSERM U1116, F‐CRIN INI‐CRCT (Cardiovascular and Renal Clinical Trialists) Nancy France; ^10^ Department of Medical specialties and Nephrology‐Hemodialysis Princess Grace Hospital, Monaco, and Centre d'Hémodialyse Privé de Monaco Monaco

**Keywords:** chronic kidney disease, diabetes, finerenone, hyperkalemia, potassium

## Abstract

**Introduction:**

Finerenone, a selective nonsteroidal mineralocorticoid receptor antagonist, has favourable effects on cardiorenal outcomes in patients with mild‐to‐severe chronic kidney disease with increased albuminuria and type 2 diabetes.

**Methods:**

Two large, randomized trials have evaluated the effects of finerenone on clinical outcomes. The first trial (FIDELIO‐DKD) investigated renal outcomes, and the second (FIGARO‐DKD) cardiovascular outcomes.

**Results:**

Patients in the two studies had a high intrinsic risk of hyperkalemia due to type 2 diabetes, treatment with optimized doses of an inhibitor of the renin‐angiotensin system, and, in some patients, their advanced chronic kidney disease. This was reflected in the incidence of hyperkalemia in the placebo group during the trials. Patients on finerenone had a significantly higher incidence of hyperkalemia compared with patients on placebo, but treatment discontinuation due to hyperkalemia was low, and no patients experienced death attributable to hyperkalemia. Structured routine potassium monitoring with temporary treatment interruption and dose reduction, as used in the two trials, should ensure the safe use of finerenone to protect the kidneys and cardiovascular system of patients with albuminuric chronic kidney disease and type 2 diabetes.

**Conclusions:**

The aim of this document is to highlight the routine potassium management required when using finerenone and to provide practical recommendations.

## Graphical abstract

Finerenone, a selective nonsteroidal mineralocorticoid receptor antagonist, has favorable effects on cardiorenal outcomes in patients with mild to severe chronic kidney disease with increased albuminuria and type 2 diabetes. This article highlights the routine potassium management required when using finerenone and provides furthermore practical recommendations.

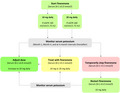

## 1 INTRODUCTION

Finerenone is a novel nonsteroidal selective antagonist of the mineralocorticoid receptor (MR), which is activated by aldosterone and cortisol.^1^ Furthermore, finerenone has high potency and selectivity for the MR. Finerenone blocks MR‐mediated sodium reabsorption and MR overactivation as well as modifies tissue remodelling by exerting anti‐inflammatory, antifibrotic, and antiproliferative effects on both the kidney and the heart.^2^, ^3^ The FInerenone in reducing kiDnEy faiLure and dIsease prOgression in Diabetic Kidney Disease (FIDELIO‐DKD)^4^ and FInerenone in reducinG cArdiovascular moRtality and mOrbidity in Diabetic Kidney Disease (FIGARO‐DKD)^5^ phase III trials evaluated the effect of the novel mineralocorticoid receptor antagonist (MRA) finerenone. These trials investigated the efficacy and safety of finerenone, on top of maximum tolerated labelled dose of an angiotensin‐converting enzyme inhibitor (ACEi) or angiotensin receptor blocker (ARB), on kidney and cardiovascular outcomes in patients with mild‐to‐severe chronic kidney disease (CKD) and type 2 diabetes (T2D). The FIDELIO‐DKD trial was designed to demonstrate a treatment effect of finerenone on kidney end‐points, whereas the FIGARO‐DKD trial was designed to demonstrate an effect on a composite cardiovascular primary end‐point. Both trials included a broad range of patients with CKD and T2D.^4^, ^5^ The FIDELITY analysis with the complementary FIDELIO‐DKD and FIGARO‐DKD trials performed a pooled analysis of efficacy and safety at the individual patient level across a broad spectrum of CKD to provide more robust estimates of the safety and efficacy of finerenone compared with placebo.^6^

In its inherent mode of action as an MRA, finerenone may elevate serum potassium concentrations, particularly in patients with advanced CKD receiving the maximum tolerated dose of an inhibitor of the renin‐angiotensin system (RAS). Both studies showed increased serum potassium concentrations and hyperkalemia rates with finerenone treatment compared with placebo, with a maximum difference in mean serum potassium between groups of 0.16 mmol/L in FIDELIO‐DKD and 0.23 mmol/L in FIGARO‐DKD. The increase in risk of hyperkalemia leading to permanent treatment discontinuation (1.7% for finerenone and 0.6% for placebo) was small.^4^, ^5^, ^6^

In this article, we aim to highlight aspects of potassium management with finerenone that were applied in the two trials and that are of particular relevance to clinical practice. We believe that a simple strategy for the management of hyperkalemia is important among clinicians, when several renoprotective drugs (e.g., RAS blockers, finerenone) may cause increases in potassium concentrations. Consequently, there is a high risk of drug discontinuation, with an increased risk of progression towards end‐stage kidney disease (ESKD). This discontinuation can be avoided by applying simple protocols for potassium management. In this regard, we spotlight the fact that changes in patient's serum potassium concentrations were predictable and manageable with routine potassium monitoring throughout the entire finerenone trial program. This reliable routine may thereby serve as a basis for potassium management with finerenone in clinical practice.

## 2 SCIENTIFIC BACKGROUND

### 2.1 Definition of hyperkalemia

Hyperkalemia can be classified as acute (as occurring in an emergent setting), chronic, or recurrent, depending on the onset and number of hyperkalemia episodes that have occurred.^7^ The decision of whether emergency therapy is warranted is largely based on subjective clinical judgement. The lack of robust and evidence‐based treatment guidelines for the management of hyperkalemia in the emergency department poses a challenge for treatment.^8^

Although the European Society of Cardiology (ESC),^9^ Kidney Disease: Improving Global Outcomes (KDIGO),^10^ and other organizations, such as the American College of Cardiology (ACC), American Heart Association (AHA), and Hearth Failure Society of America (HFSA)^11^ have issued guidelines, the concentration of potassium that is labelled as hyperkalemia varies. Serum potassium concentrations of 5.0,^11^ 5.5,^12^ or 6.0 mmol/L^13^ are commonly used cutoffs for the definition of hyperkalemia.

The KDIGO controversies' conference report defines the severity of hyperkalemia by both serum potassium concentration and ECG changes.^10^ The most common ECG change is peaked T waves, followed by QRS prolongation. Hyperkalemia is classified as mild, moderate, or severe based on potassium concentration and the presence or absence of ECG changes. Serum potassium concentrations ≥5.0–5.9 mmol/L are typically defined as mild and ≥6.0–6.4 mmol/L as moderate. Serum potassium concentrations ≥6.5 mmol/L are typically defined as severe.^10^

### 2.2 Risk factors of hyperkalemia

Abnormal serum potassium concentrations represent one of the most important electrolyte disorders in clinical practice. In this regard, potassium plays a critical role in normal cell membrane electrophysiology, with both hyperkalemia and hypokalemia leading to electrophysiological perturbations.^14^ While mild hyperkalemia is usually asymptomatic, high potassium concentrations can cause life‐threatening cardiac arrhythmias, muscle weakness, or paralysis. The kidneys play a central role in potassium homeostasis. Kidney dysfunction and acquired or inherited defects in potassium excretion in the distal nephron are among the most important risk factors for hyperkalemia.^15^ Accordingly, the main risk factors for hyperkalemia are kidney failure, diabetes mellitus, and adrenal disease.^16^

An international meta‐analysis of over 1.2 million patients with CKD from 27 different cohorts examined the risk factors leading to hyperkalemia. The risk of hyperkalemia correlated strongly with the estimated glomerular filtration rate (eGFR): a decrease in eGFR of 15 ml/min per 1.73 m^2^ approximately doubled the risk of hyperkalemia.^17^ While lower eGFR was a strong risk factor for hyperkalemia, higher albuminuria was a weaker risk factor.^17^ Furthermore, medications such as potassium‐sparing diuretics, MRAs, ACE inhibitors, ARBs, direct renin antagonists, β‐blockers, nonsteroidal anti‐inflammatory drugs, heparin, and penicillin are associated with hyperkalemia.^18^ Potassium supplements and potassium‐based salt substitutes may also be a cause of hyperkalemia, especially in patients with underlying CKD or concomitant use of hyperkalemia‐inducing drugs.^19^

## 3 FINERENONE CLINICAL OUTCOMES

### 3.1 FIDELIO‐DKD

The FIDELIO‐DKD^4^ trial included 5734 patients with T2D and CKD. Patients were required to have persistent high albuminuria (UACR ≥30 but <300 mg/g) with an eGFR ≥25 to ≤60 ml/min/1.73 m^2^ and a history of diabetic retinopathy or severe albuminuria (UACR ≥300 but <5000 mg/g) and an eGFR ≥25 to ≤75 ml/min/1.73 m^2.^ ^4^ All patients were treated with RAS blockade at the maximum tolerated dose. The median follow‐up time was 2.6 years. At screening, 640/7114 (9.0%) patients had serum potassium concentrations >4.8 mmol/L and dropped out of the screening. At baseline, the mean serum potassium concentration was 4.37 ± 0.46 mmol/L in the finerenone group and 4.38 ± 0.46 mmol/L in the placebo group.^20^ Hyperkalemia was a treatment‐emergent, investigator‐reported adverse event. Hyperkalemia was considered serious if it resulted in death, was life‐threatening, required inpatient hospitalization, caused persistent or significant disability or incapacity, or was judged by the investigator to be a serious or important medical event (Table 1).^4^

**TABLE 1:** FIDELIO‐DKD clinical outcomes^4^

FIDELIO‐DKD—finerenone vs placebo for CKD outcomes in patients with T2D and CKD
Primary outcome: composite of kidney failure, a sustained decrease of at least 40% in the eGFR from baseline for at least 4 weeks, or death from renal causes

Outcome	Finerenone	Placebo	HR (95% CI), *p*‐value
Primary composite outcome	17.8%	21.1%	0.82 (0.73–0.93), *p* = .001
Kidney failure	7.3%	8.3%	0.87 (0.72–1.05)
GFR decrease ≥40%	16.9%	20.3%	0.81 (0.72–0.92)
Death from renal causes	<0.1%	<0.1%	N/A
Overall mortality	7.7%	8.6%	HR 0.90, 95%CI [0.75–1.07]
Hyperkalemia	18.3%	9%	N/A
Serious hyperkalemia	1.6%	0.4%	N/A
Hospitalizations related to hyperkalemia	1.4%	0.3%	N/A
Hyperkalemia leading to permanent discontinuation	2.3%	0.9%	N/A
Deaths attributable to hyperkalemia	0%	0%	N/A

In patients with CKD and T2D, treatment with finerenone resulted in lower risks of CKD progression and cardiovascular events than placebo.

### 3.2 FIGARO‐DKD

The FIGARO‐DKD trial included 7437 patients with T2D and CKD.^5^ Patients were required to have persistent high albuminuria (UACR ≥30 but <300 mg/g) with an eGFR ≥25 to ≤90 ml/min/1.73 m^2^ and a history of diabetic retinopathy or severe albuminuria (UACR ≥300 but <5000 mg/g) and an eGFR of at least 60 ml/min/1.73 m^2^. All patients were treated with RAS blockade at the maximum tolerated dose. The median follow‐up time was 3.4 years.^5^ Patients were required to have a serum potassium concentration of 4.8 mmol/L or less at the time of screening. As in FIDELIO‐DKD, hyperkalemia was a treatment‐emergent, investigator‐reported adverse event (Table 2).

**TABLE 2:** FIGARO‐DKD clinical outcomes^5^

FIGARO‐DKD—finerenone vs placebo for CVD outcomes in patients with T2D and CKD
Primary outcome: Composite of death from cardiovascular causes, nonfatal myocardial infarction, nonfatal stroke, or hospitalization for heart failure

Outcome	Finerenone	Placebo	HR (95% CI), *p*‐value
Primary composite outcome	12.4%	14.2%	0.87 (0.76–0.98), *p* = .03
Death from CV causes	5.3%	5.8%	0.90 (0.74–1.09]
Nonfatal myocardial infarction	2.8%	2.8%	0.99 (0.76–1.31)
Nonfatal stroke	2.9%	3.0%	0.97 (0.74–1.26)
Hospitalization for heart failure	3.2%	4.4%	0.71 (0.56–0.90)
Overall mortality	9.0%	10.1%	0.89 (0.77–1.04)
Hyperkalemia	10.8%	5.3%	N/A
Serious hyperkalemia	0.7%	0.1%	N/A
Hospitalizations related to hyperkalemia	0.6%	0.1%	N/A
Hyperkalemia leading to permanent discontinuation	1.2%	0.4%	N/A
Deaths attributable to hyperkalemia	0%	0%	N/A

In patients with T2D and stage 2 to 4 CKD with moderately increased albuminuria or stage 1 or 2 CKD with severely increased albuminuria, finerenone improved cardiovascular outcomes compared with placebo.

### 3.3 FIDELITY

FIDELITY, a pooled‐analysis of more than 13,000 patients from the FIGARO‐DKD and FIDELIO‐DKD phase III trials, demonstrated cardiovascular and kidney benefits of finerenone in patients with CKD and T2D.^6^ In the FIDELITY analysis, finerenone reduced the risk for the composite cardiovascular end‐point, i.e., time to cardiovascular death, nonfatal myocardial infarction, nonfatal stroke, or hospitalization for heart failure, by 14% compared with placebo (HR:0.86 [95% CI: 0.78–0.95]; *p* = .0018). The risk of composite kidney event, i.e., time to the first episode of kidney failure, sustained decrease in eGFR by ≥57% from baseline over a period of ≥4 weeks, or death from kidney failure, was 23% lower with finerenone compared with placebo (HR: 0.77 [95% CI: 0.67–0.88]; p = 0.0002). A kidney‐related event occurred in 360 (5.5%) patients on finerenone and 465 (7.1%) on placebo.^6^ Although overall incidence of hyperkalemia was low, low eGFR was associated with higher incidence of hyperkalemia leading to discontinuation or hospitalization (Table 3).

**TABLE 3:** Treatment‐emergent hyperkalaemia events

Treatment‐emergent hyperkalemia events	eGFR <60 ml/min/1.73 m^2^		eGFR ≥60 ml/min/1.73 m^2^	
	Finerenone (*n* = 3908)	Placebo (*n* = 3900)	Finerenone (*n* = 2602)	Placebo (*n* = 2588)
Any	714 (18.3%)	333 (8.5%)	198 (7.6%)	115 (4.4%)
Leading to permanent discontinuation	94 (2.4%)	31 (0.8%)	16 (0.6%)	7 (0.3%)
Leading to hospitalization	53 (1.4%)	10 (0.3%)	8 (0.3%)	0 (0%)

## 4 POTASSIUM MANAGEMENT AND POTASSIUM CONCENTRATIONS IN FIDELIO‐DKD and FIGARO‐DKD

In the FIDELIO‐DKD and the FIGARO‐DKD trials, hyperkalemia was reported using thresholds of >5.5 mmol/L for mild hyperkalemia and >6.0 to <6.5 mmol/L for moderate hyperkalemia, in accordance with the latest KDIGO guidance based on the protocol.^10^ The earliest time point after which serum potassium was measured in both trials was 1 month after treatment initiation. The second scheduled assessment of serum potassium was at the fourth month after treatment initiation and at 4‐month intervals thereafter.^4^, ^5^ The frequency of potassium monitoring in both trials was consistent with that recommended in the KDIGO guidelines for patients with CKD (3–4 times per year for patients with UACR >300 mg/g and eGFR <60 ml/min per 1.73 m^2^ and 2–3 times per year for patients with UACR 30–300 mg/g and eGFR 15–59 ml/min per 1.73 m^2^).^10^ In both trials, the risk of hyperkalemia from finerenone (14.0%) versus placebo (6.9%) was more than doubled.^4^, ^5^, ^6^ This occurred despite the fact that potassium‐increasing drugs other than ACE inhibitors and ARBs were prohibited and subjects with baseline potassium concentrations >4.8 mmol/L were excluded from the trials.^4^, ^5^

Finerenone had a predictable impact on serum potassium concentrations in both trials with a maximum difference in mean serum potassium between groups of 0.23 mmol/L in FIDELIO‐DKD and 0.16 mmol/L in FIGARO‐DKD. Given the higher mean eGFR in the FIGARO‐DKD trial compared with the FIDELIO‐DKD trial (68 vs. 44 ml/min/1.73 m^2^), the incidence of hyperkalemia with finerenone treatment was lower in the former (10.8% vs. 18.3%), despite the longer median follow‐up (3.4 vs. 2.6 years).^4^, ^5^ Of note, a post‐hoc analysis of the FIDELIO‐DKD data showed that independent risk factors for mild hyperkalemia were higher serum potassium, lower eGFR, increased urine albumin/creatinine ratio, younger age, female sex, ß‐blocker use, and finerenone assignment. Diuretic or SGLT2 inhibitor use reduced risk.^20^

Although hyperkalemia was increased in both trials, the incidence of hyperkalemia‐related adverse events with clinical impact was low, with hyperkalemia‐related permanent treatment discontinuation in only 1.7% of patients receiving finerenone versus 0.6% with placebo.^6^ Potassium intake was not restricted during the trials, but finerenone or placebo was withheld in cases where potassium concentrations >5.5 mmol/L were detected until potassium concentrations fell to <5.0 mmol/L. With a robust potassium management strategy guided by regular monitoring of serum potassium, there were no hyperkalemia‐related deaths in more than 13,000 patients over a median follow‐up of 3 years.^6^

### 4.1 Recommendations for the management of potassium with Finerenone

**FIGURE 1:**
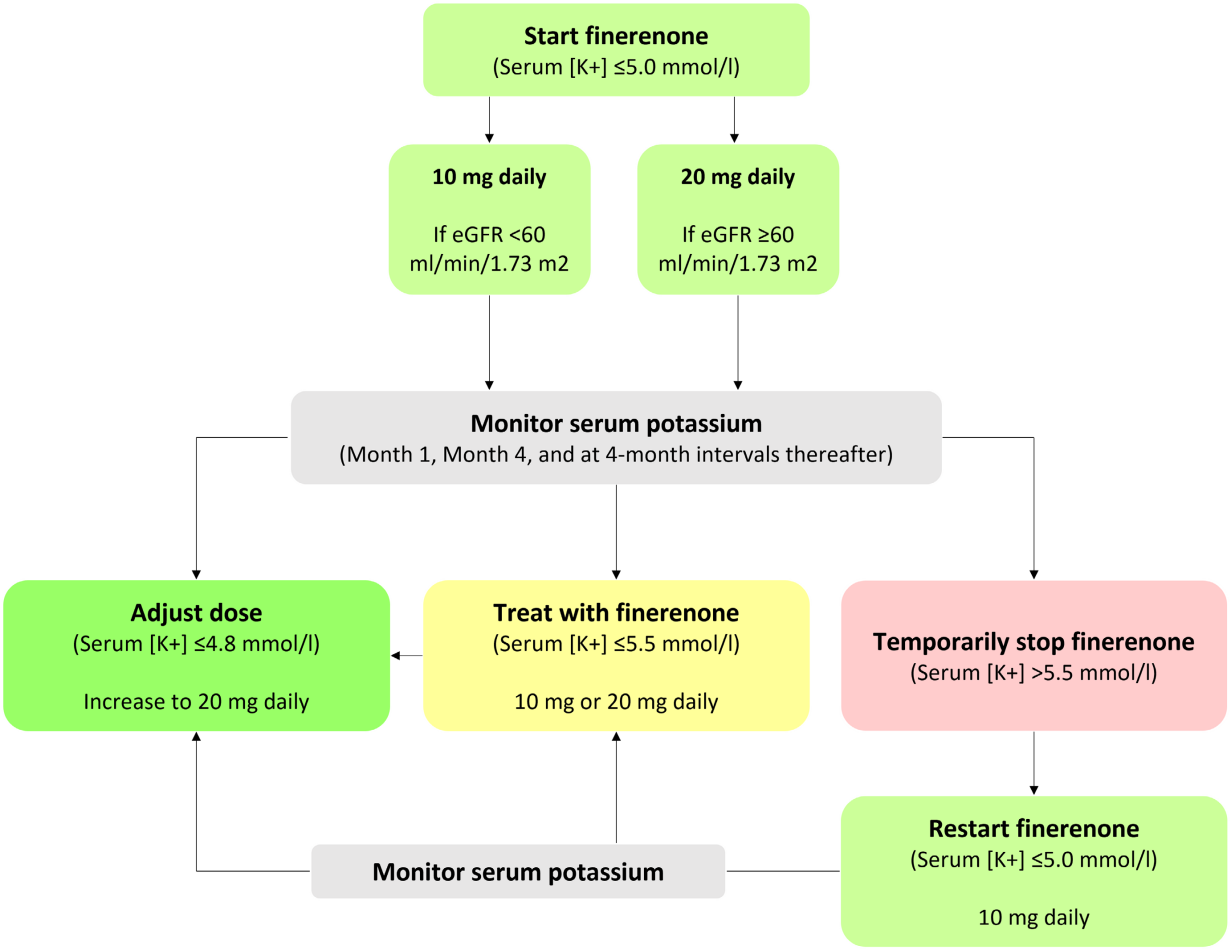
Protocol for potassium management

In accordance with the US labelling prescribing information for finerenone, serum potassium and eGFR should be measured and finerenone should be dosed appropriately in all patients prior to initiation of treatment.^21^ Treatment with finerenone should not be initiated if serum potassium is >5.0 mmol/L. Serum potassium should be monitored periodically, on a schedule similar to study visits in FIDELIO‐DKD and FIGARO‐DKD (Month 1, Month 4, and at 4‐month intervals thereafter) to minimize the risk of hyperkalemia.^16^ Importantly, such a schedule is actually similar with the usual biological monitoring recommended in CKD patients by the KDIGO 2012 guidelines (Figure 1).^10^

If serum potassium concentrations are ≤4.8 mmol/L, the dose should be continued or increased to 20 mg per day (if the starting dose was 10 mg per day, i.e., for patients with a baseline eGFR 25 to <60 ml/min/1.73 m^2^). If serum potassium concentrations are >4.8‐≤ 5.5 mmol/L, the dose should be maintained at 10 mg daily or 20 mg daily.^21^ Serum potassium should be monitored 4 weeks after a dose adjustment and throughout treatment, and the dose adjusted as needed.

This is actually similar to the recommended procedure for RASi oversight, per KDIGO guidelines on diabetes management in CKD, which state “Monitor serum creatinine and potassium (within 2–4 weeks after starting or changing dose)”.^22^ Finerenone should be temporarily discontinued if serum potassium concentrations are >5.5 mmol/L and can be restarted at 10 mg daily when serum potassium concentrations are ≤5.0 mmol/L. Finerenone has a short half‐life of 2–3 h and no active metabolites; therefore, hyperkalemia can be treated by discontinuing treatment.^23^ For patients with moderate liver disease (Child‐Pugh B), additional potassium monitoring should be considered (Table 4).^21^

**TABLE 4:** Dose adjustment based on current serum potassium concentration and current dose^21^

Dose adjustment based on serum potassium		
	Current finerenone dose	
Potassium concentration (mmol/l)	10 mg	20 mg
≤ 4.8	Increase the dose to 20 mg once daily	Maintain 20 mg dose daily
> 4.8 ‐ ≤ 5.5	Maintain 10 mg once daily	Maintain 20 mg once daily
> 5.5	Withhold finerenone Consider restarting at 10 mg once daily when serum potassium ≤5.0 mmol/L	Withhold finerenone Restart at 10 mg once daily when serum potassium ≤5.0 mmol/L

### 4.2 Practical considerations to minimize the risk of hyperkalemia with finerenone

Patients at the highest risk of hyperkalemia are those with low eGFR and high baseline serum potassium concentrations. More frequent serum potassium monitoring may be required for higher‐risk patients, including those using medications that impair potassium excretion or increase serum potassium concentrations.^16^ Trigger events for hyperkalemia may include a potassium‐rich diet, acute sickness (e.g., acute kidney injury, volume depletion, GI problems, and infection), new comedications such as nonsteroidal anti‐inflammatory drugs (NSAIDS), and surgery.^4^, ^5^, ^24^

Finerenone is a CYP3A4 substrate. Concomitant use with a CYP3A4 inhibitor increases finerenone exposure, which may increase the risk of adverse reactions.^25^ The concomitant use of finerenone with itraconazole, clarithromycin, and other strong CYP3A4 inhibitors (e.g., ketoconazole, ritonavir, nelfinavir, cobicistat, telithromycin, or nefazodone) is contraindicated. Concomitant intake of grapefruit or grapefruit juice should be avoided.^26^ Concomitant use with a moderate CYP3A4 inhibitor (such as erythromycin and verapamil) or a weak CYP3A4 inhibitor (such as fluvoxamine) requires serum potassium monitoring during initiation of treatment or dose adjustment of finerenone or the moderate or weak CYP3A4 inhibitor and appropriate finerenone dosage adjustment.^21^ Furthermore, finerenone should not be used concomitantly with rifampicin and other strong CYP3A4 inducers (e.g., carbamazepine, phenytoin, phenobarbital, St. John's wort) or with efavirenz and other moderate CYP3A4 inducers. These CYP3A4 inducers are expected to decrease the plasma concentration of finerenone, resulting in an attenuation of the therapeutic effect.^21^, ^25^

When combined with other potassium‐raising medications, the risk of hyperkalemia is increased.^16^ Finerenone should not be used concomitantly with potassium‐sparing diuretics (e.g., amiloride, triamterene) and other MRAs (e.g., eplerenone, esaxerenone, spironolactone, and canrenone).^21^ More frequent serum potassium monitoring is warranted if finerenone is coadministered with drugs or supplements that increase serum potassium or impair potassium excretion.^21^ Coadministration of potassium‐wasting diuretics and potassium binders can be considered, as they reduce serum potassium concentrations.^7^ Of note, potassium binder use was uncommon at investigator discretion in FIDELIO‐DKD (10.8% with Finerenone vs. 6.5% with placebo) and in FIGARO‐DKD (4.5% with Finerenone vs. 2.8% for placebo).^4^, ^5^ Sodium bicarbonate can be used to correct metabolic acidosis.^27^

### 4.3 Practical considerations for dietary intake

If serum potassium is elevated with normal acid–base balance, dietary modification is recommended to reduce potassium intake from foods of lower nutritional value after other non‐nutritional causes such as medications have been considered and treated, if medically appropriate.^28^ Many fresh fruits, and vegetables such as bananas, oranges, melon, honeydew, apricots, and grapefruit are rich in potassium.^29^ Some dried fruits such as prunes, raisins, and dates are also rich in potassium. In addition, potassium is also found in many beverages such as fruit juices and coconut water. Meat, fish, and dairy foods often contribute more dietary potassium than fruits and vegetables, so consideration of the dietary pattern or a whole‐diet plan is required for optimal management.^28^ If hyperkalemia does occur, patients should avoid salt substitutes and receive nutritional counselling to reduce excessive potassium intake.^7^, ^29^

In a study comparing high consumption of fruits and vegetables against sodium bicarbonate and control conditions, fruit and vegetable consumption was as effective as sodium bicarbonate in reducing acidosis and slowing the decline in eGFR without increasing serum potassium.^30^ In addition, high fruit and vegetable consumption was superior to sodium bicarbonate in lowering body weight, systolic blood pressure, and low‐density lipoprotein (LDL) cholesterol.^30^ It is important to note that dietary modifications are now recommended only to treat hyperkalemia and not as a preventative measure.^28^ When possible, individuals with CKD should be encouraged to eat a variety of plant foods for dietary fibre, cardio‐protection, and the beneficial effect on gut microbiome. In the past decade, there has been a paradigm shift in the nutritional management of CKD. The focus has moved away from management of specific nutrients and towards the broader perspective of whole diets and dietary patterns.^28^

Table 5 shows the current recommendations from the 2020 KDIGO Guidelines for Diabetes in CKD^22^ and 2020 Kidney Disease Outcomes Quality Initiative (KDOQI) Guidelines for nutrient requirements in adult patients with CKD.^31^ A caloric intake of 25–35 kcal/kg/day is recommended to compensate for excessive resting energy expenditure due to inflammation and comorbidities and to maintain a neutral or positive nitrogen balance. However, this recommendation should be individualized to the patient's profile, including age, lean body mass, physical activity, and underlying aetiology of kidney disease.^31^

**TABLE 5:** Nutritional requirements for patients with CKD according to 2020 KDIGO and KDOQI Guidelines^22^, ^31^

Energy (kcal/kg ideal weight/day)^a^	25–35 kcal
Protein (g/kg/day)^a^	0.55–0.60 g 0.80 g (patients with diabetes and non‐dialysis CKD) 1.0–1.2 g (patients with haemodialysis)
Potassium^b^	Adjust dietary potassium intake to maintain serum potassium within the normal range
Sodium (g/day)	<2.0 g
Calcium (mg/day)^c^	800–1000 mg
Phosphorus^d^	Adjust dietary phosphorus intake to maintain serum phosphate levels in the normal range
Fibre (g/day)	25–38 g
Vitamin D (IU/day)	600–800 IU

^a^ Energy and protein intake should be adapted to age, gender, level of physical activity, body composition, weight status goals, CKD stage, and concurrent illness or presence of inflammation to maintain normal nutritional status. If present, priority should be given to the correction of protein‐energy wasting.

^b^ Guidelines do not suggest specific dietary K range. Before restricting healthy foods, other causes of hyperkalemia should be corrected.

^c^ Including dietary calcium, calcium supplementation, and calcium‐based phosphate/potassium binders.

^d^ When making decisions about phosphorus restriction treatment, consider the bioavailability of phosphorus sources.

The recommended protein intake for stable patients with CKD is 0.55–0.60 g/kg/day, which can be reduced to 0.28–0.43 g/kg/day if it is supplemented with 7–15 g/day of KAs and essential AAs.^31^ For patients with diabetes, the 2020 KDIGO guidelines suggest maintaining a higher protein intake up to 0.8 g/kg/day to glycemic control. Substantial sodium restriction (<2 g/day) is recommended to achieve better volume control, lower blood pressure, and treat proteinuria synergistically with available pharmacologic interventions.^22^ However, a daily fibre intake of 25–30 g/day or more may also be recommended for patients with CKD, which is the recommended amount for the general population. In general, patients with CKD do not require aggressive dietary potassium restriction until advanced stages or when the risk of hyperkalemia is considered high.^31^

Patients should not provide breastfeeding while using finerenone and for 1 day after stopping treatment. Patients with severe hepatic impairment should not take finerenone. Patients with moderate hepatic impairment may require additional serum potassium monitoring.^21^

## 5 CONCLUSION

Finerenone shows kidney and cardiovascular benefits across the spectrum of patients with chronic kidney disease and type 2 diabetes, and it is well tolerated. Finerenone was associated with a low absolute risk of clinically relevant hyperkalemia, with only a small proportion of patients discontinuing treatment due to hyperkalemia, and no deaths attributed to hyperkalemia. A validated protocol for potassium monitoring and hyperkalemia management should be applied to enhance safety and to enable successful management of CKD with finerenone.

## AUTHOR CONTRIBUTIONS

**Christoph Wanner:** Conceptualization (lead); writing – review and editing (lead). **Paola Fioretto:** Writing – review and editing (equal). **Csaba P Kovesdy:** Writing – review and editing (equal). **Jolanta Malyszko:** Writing – review and editing (equal). **Roberto Pecoits‐Filho:** Writing – review and editing (equal). **Oliver Schnell:** Writing ‐ review and editing (equal). **Patrick Rossignol** Writing ‐ review and editing (equal).

## FUNDING INFORMATION

The publication has been funded by an unrestricted Educational Grant from Bayer AG.

## CONFLICT OF INTEREST

Dr. Wanner received consulting fees and honoraria for lecturing from AstraZeneca, Bayer, Boehringer‐Ingelheim, FMC, GILEAD, GSK, Lilly, MSD, Sanofi and Vivor. Dr. Fioretto received condultation fees from AstraZeneca, Bayer, Boehringer‐Ingelheim, Lilly, Mundipharma, NovoNordisk. Dr. Kovesdy received consulting fees from Abbott, Akebia, AstraZeneca, Bayer, CSL Behring, Boehringer Ingelheim, Rockwell and Vifor. Dr. Rossignol: consulting for AstraZeneca, Bayer, Boehringer‐Ingelheim, CinCor, G3P, Idorsia, KBP, Novartis, Novo Nordisk, Relypsa Inc., a Vifor Pharma Group Company, Roche, Sanofi, Sequana Medical, Servier, Tasso, Vifor Fresenius Medical Care Renal Pharma, Vifor; Cofounder: CardioRenal, a company developing potassium and creatinine sensors for home self‐monitoring. Dr. Malyszko received consulting fees from AstraZeneca, Bayer, Servier. Dr. Oliver Schnell is a founder and CEO of Sciarc GmbH. Dr. Pecoits‐Filho: Research grants from Fresenius Medical Care, National Council for Scientific and Technological Development, grants (paid to employer) from Astra Zeneca, Boehringer‐Lilly, Novo Nordisk, Akebia, Bayer for participation in advisory Boards and educational activities. RPF is employed by Arbor Research Collaborative for health, who runs the DOPPS studies. Global support for the ongoing DOPPS Programs is provided without restriction on publications by a variety of funders. Funding is provided to Arbor Research Collaborative for Health and not to Dr. Pecoits‐Filho directly. For details, see https://www.dopps.org/AboutUs/Support.aspx

## Data Availability

Data sharing is not applicable to this article as no new data were created or analyzed in this study.
